# Pilot ED Wastewater Surveillance During the 2024-2025 Respiratory Virus Season

**DOI:** 10.1001/jamanetworkopen.2025.55023

**Published:** 2026-01-22

**Authors:** Zachary Renfro, Alessandro Zulli, Vivian Levy, Roma Nawy, Alicia Mercado, Sehee Jong, Jorge Luis Salinas, Heather Eastwood, Chad Below, Alexandria B. Boehm, Julie Parsonnet, Christopher L. Bennett

**Affiliations:** 1 Department of Emergency Medicine, Stanford University School of Medicine, Stanford, California; 2 Department of Civil & Environmental Engineering, Stanford University, Stanford, California; 3 San Mateo County Health, San Mateo, California; 4 Division of Infectious Diseases & Geographic Medicine, Department of Medicine, Stanford University School of Medicine, Stanford, California

## Abstract

This cross-sectional study compares emergency department (ED) wastewater surveillance for prevalence of respiratory viral pathogens with clinical testing trends at 2 urban US hospitals during the 2024-2025 respiratory virus season.

## Introduction

Infectious disease surveillance typically relies on clinical case reports. These systems are prone to reporting lags and often fail to fully capture changes in disease incidence or prevalence. Wastewater surveillance offers a complementary approach to conventional methods. Wastewater surveillance efforts in the US currently focus on sampling conducted at wastewater treatment plants.^1^ Emergency departments (EDs) represent sentinel care venues during outbreaks and for emerging pathogens, serving as safety nets for vulnerable populations without alternative sources of care.^2^ ED-based wastewater monitoring might provide more detailed geographic insights that could help guide timely and targeted interventions.

## Methods

This cross-sectional study was deemed exempt from approval and informed consent by the Stanford University institutional review board because it was not human participants research; it followed the STROBE reporting guideline.^3^ After site assessments and impact analyses, surveillance of ED wastewater was implemented during the respiratory virus season from December 9, 2024, to May 30, 2025, at 2 urban California EDs: San Mateo Medical Center (38 783 annual visits) and Stanford University Hospital (95 094 annual visits). These EDs have different sewersheds and serve distinct populations, with differences in insurance coverage, age distribution, and patterns of health care access. At both sites, passive sampling through porous devices that absorb wastewater specific to the ED over time were deployed, obtaining samples approximately 3 times weekly, with each sampler left in place for a 48-hour interval.^4^

Nucleic acids were extracted from samples and pathogen concentrations were measured using reverse-transcription droplet digital polymerase chain reaction (RT-ddPCR). Target viruses included SARS-CoV-2 (N gene), influenza A (types H1, H3, and H5), influenza B, respiratory syncytial virus (RSV), and mpox (clade I E3L).^5^ Analysis incorporated a negative nonviral control, a positive bovine coronavirus process control, and an endogenous pepper mild mottle virus control to account for variation in fecal volume and concentration in samples. A sample was considered positive for a pathogen if nucleic acid was detected in at least 3 droplets across replicate RT-ddPCR wells. In parallel, weekly counts of positive ED diagnostic tests for SARS-CoV-2, influenza A and B, RSV, and mpox were obtained to assess whether wastewater trends visually corresponded with clinical testing trends. Analysis and visualization were performed in RStudio, version 4.5.1 (RStudio, PBC).

## Results

**Figure 1.**
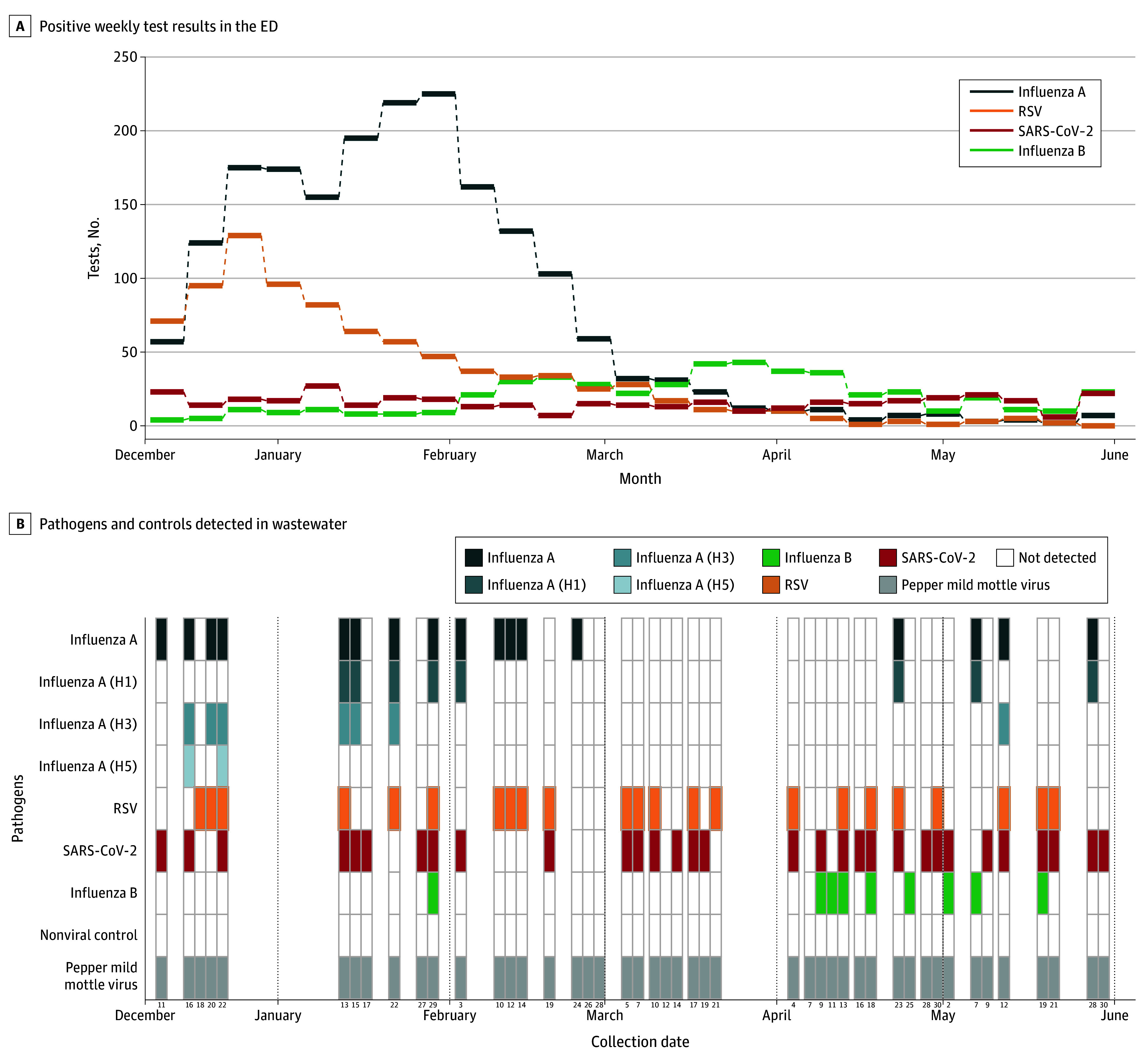
Results From a Pilot Study of Wastewater Surveillance in Stanford Hospital During the 2024-2025 Respiratory Virus Season B, The heatmap represents the binary outcome of whether a specific pathogen was amplified from an emergency department (ED) wastewater sample collected on the corresponding date. RSV indicates respiratory syncytial virus.

**Figure 2.**
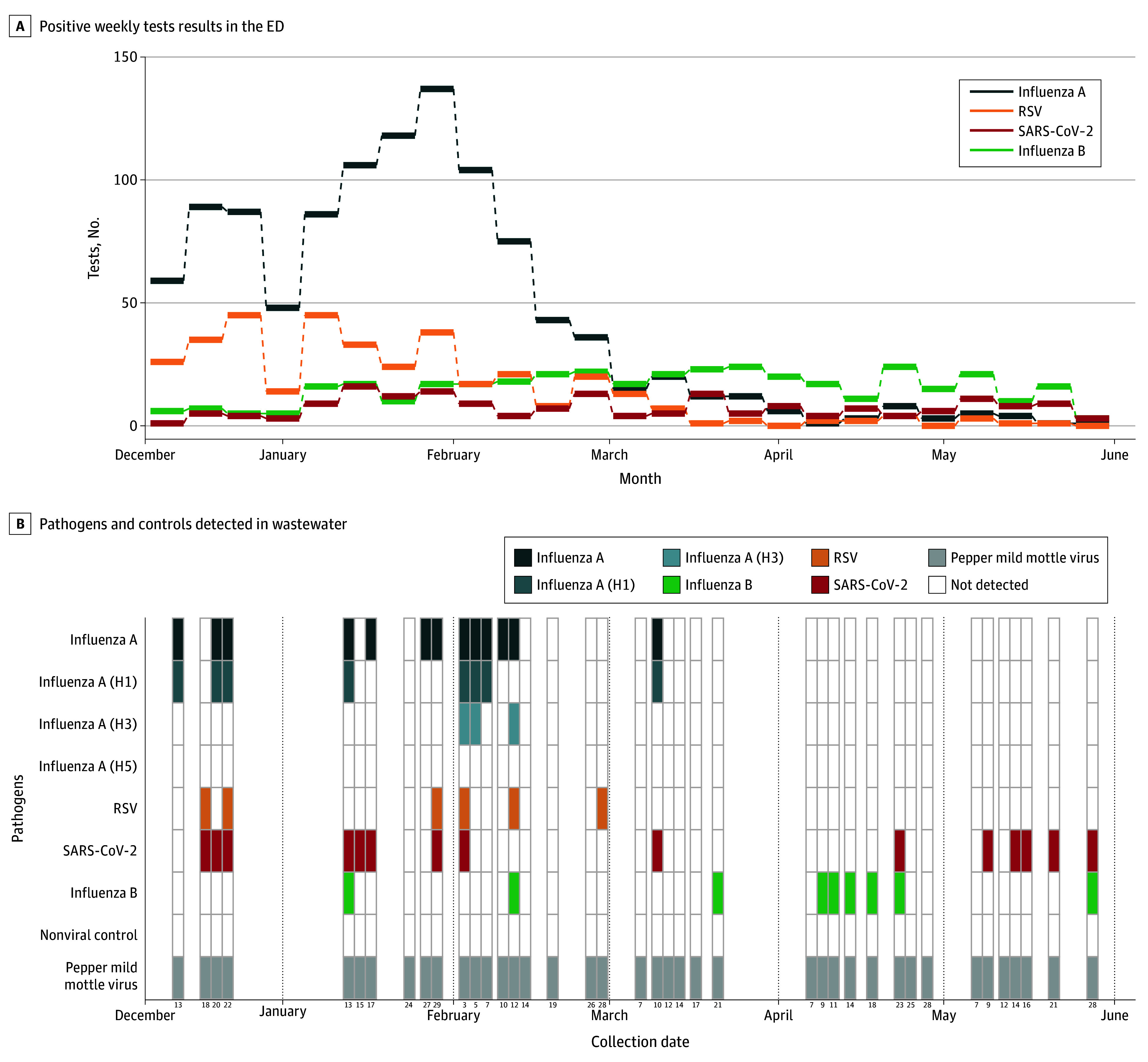
Results From a Pilot Study of Wastewater Surveillance in the San Mateo Medical Center During the 2024-2025 Respiratory Virus Season B, The heatmap represents the binary outcome of whether a specific pathogen was amplified from an emergency department (ED) wastewater sample collected on the corresponding date. RSV indicates respiratory syncytial virus.

During the study period, 86 wastewater samples were collected across the 2 sites. Nucleic acids corresponding to multiple pathogens were detected, with distinct patterns observed across locations and time (Figure 1 and Figure 2). Throughout the study period, variation between sites was broadly consistent with clinical testing, although patterns varied by pathogen and site. At both sites, SARS-CoV-2 RNA was most consistently detected (31 of 46 samples at Stanford Hospital [67%] and 15 of 40 [38%] at San Mateo). Influenza A RNA was detected in 17 samples (37%) at Stanford Hospital and 13 (32%) at San Mateo; at Stanford, this coincided with peak positive clinical test results.

## Discussion

This pilot study demonstrated the technical feasibility of ED-based wastewater surveillance for respiratory viral pathogens—an approach with unique logistic and infrastructure challenges compared with wastewater sampling at treatment plants. Across both study sites, ED wastewater showed general concordance with ED clinical testing. These findings highlight the potential value of integrating ED wastewater surveillance into broader public health monitoring, particularly in high-volume or safety-net facilities where some patients might decline testing or remain untested because of an asymptomatic infection or limited resources. The findings suggest this complementary approach can be used to identify pathogens for which testing might be underutilized (eg, viral hepatitis, integrase-resistant HIV, or drug-resistant gonorrhea). This pilot study was limited by its short time frame, small number of sites included, and constraints on directly correlating ED clinical testing with wastewater data. However, by providing department-level information that can be tied to operations, ED-based sampling offers a level of operational immediacy not available from wastewater treatment plant–based sampling.

## Notes

Supplement.
Data Sharing Statement
