# Supplementary material for: Pilot ED Wastewater Surveillance During the 2024-2025 Respiratory Virus Season
Source: JAMA Netw Open. 2026 Jan 22;9(1):e2555023. doi: 10.1001/jamanetworkopen.2025.55023 (PMC12828624; doi:10.1001/jamanetworkopen.2025.55023)
Supplement: Supplement. — Data Sharing Statement [file jamanetwopen-e2555023-s001.pdf]

Data Sharing Statement

Renfro. Pilot ED Wastewater Surveillance During the 2024-2025 Respiratory Virus Season. *JAMA Netw Open*. Published January 22, 2026. doi:10.1001/jamanetworkopen.2025.55023

Data
Data available: No

Additional Information
Explanation for why data not available: Individual patient data was not obtained
